# Phytochemical analysis and in vitro anthelmintic activity of *Imperata cylindrica* underground parts

**DOI:** 10.1186/s12906-020-03125-w

**Published:** 2020-11-06

**Authors:** Pawi Bawitlung Lalthanpuii, Kholhring Lalchhandama

**Affiliations:** Department of Life Sciences, Pachhunga University College, Aizawl, Mizoram 796001 India

**Keywords:** Anthelmintic, Medicinal plant, Roundworm, Scanning electron microscopy, Tapeworm

## Abstract

**Background:**

*Imperata cylindrica* is considered as an agricultural weed, but it is a valuable plant in the traditional medicines of Southeast Asia. In the Mizo traditional medicine of India and Myanmar, the rhizomes and roots are used as a remedy for bacterial, fungal and intestinal helminth infections.

**Methods:**

An extract of the whole underground parts was prepared in Soxhlet apparatus using chloroform as a solvent. After concentrating in a vacuum rotary evaporator, the extract was analysed using gas chromatography-mass spectrometry. Anthelmintic activity was tested in vitro against the tapeworm *Raillietina tetragona* and the roundworm *Ascaridia galli*. Scanning electron microscopy was used to examine the structural changes on the helminths after treatment with the plant extract.

**Results:**

Twenty-two compounds were identified from the plant extract out of which fatty acids were the predominant compounds. Palmitic acid was the most abundant. Bioactive phytosterols such as campesterol and stigmasterol were also detected. The plant extract was significantly effective on both the helminths and showed dose-dependent anthelmintic activity as that of albendazole. The tapeworm treated with the plant extract showed deformities on the suckers, clumping of the spines, tegumental folds and erosion of microtriches. Extensive damage was also seen on the roundworm including cuticular shrinkage, collapse of the lips, and formation of warty surface throughout the body.

**Conclusion:**

*I. cylindrica* extract effectively killed and caused detrimental effects on parasitic tapeworm and roundworm. The study therefore validates the traditional usage among the Mizo people, and guarantees further investigation on the exact compound(s) and mechanism of action.

## Background

Helminthiasis is one of the most persistent scourges of the health and welfare of humans, livestock animals and wildlife. It remains the major factor of human debility, poverty, cognitive weakness and sometimes death [1], as well as of huge economic losses in animal industry [2]. According to the current estimate, roundworms alone infect 1.5 billion people worldwide [3], while schistosomes (flukes) account for 220 million cases [4]. Tapeworms are the least prevalent among them but are responsible for the worst outcomes such as neurocysticercosis, which cause permanent brain damage and premature fatality. Helminthiasis also intensifies other infections such as those of *Plasmodium falciparum*, human immunodeficiency virus (HIV), and *Mycobacterium tuberculosis* [5]. No new drugs have been developed for several decades [6], while anthelmintic resistance is evolving at an accelerated pace in the most important helminth parasites of livestock animals [7] and humans [8].

The tapeworm *Raillietina tetragona* Molin, 1858, and the roundworm *Ascaridia galli* Schrank, 1788, are the most prevalent and thereby the most important parasites of birds in different parts of the world [9–11]. They cause detrimental health conditions and pathological symptoms including anaemia, droopiness, emaciation and diarrhoea [12]. The consequences such as loss of appetite, weight loss, reduced egg production and death lead to economic losses in poultry farming [13]. In spite of their huge influences in avian health and poultry production, there are no prescription anthelmintic drugs.

Medicinal plants are as a source of many important pharmaceutical drugs, but they have not yet produced a single anthelmintic drug. An interesting plant is *Imperata cylindrica* (L.) Raeusch. (family - Poaceae), the underground parts (rhizomes and roots) of which are used in Mizo traditional medicine as a treatment for intestinal helminthiasis, fungal infections, blood urine (haematuria), blood vomit (haematemesis), and nosebleed (epistaxis) [14]. Although the plant is regarded as a noxious weed [15], it is recognised in Southeast Asian cultures as an antibacterial, anticoagulant (styptic), antipyretic (febrifuge), water pill (diuretic), skin smoothening (emollient), salivating (sialagogue), and blood soothing (tonic) agent [16]. Its effects on circulatory system such as in vasodilation and blood flow have been reported [17, 18].

Practically no attention has been given to this plant in terms of chemical analysis and biological activities against parasites. It is therefore challenging to study its effect on intestinal parasites.

A remarkable fact is that while most anthelmintic drugs are helminth specific, i.e. they are effective only against a particular group of helminths, *I. cylindrica* is acclaimed to be equally effective against both tapeworms and roundworms [14]. Identification of important compounds present in the plant extract and anthelmintic tests using both tapeworm and roundworm will be useful for further investigations on the pharmacology of this plant.

## Methods

### Plant material and extraction

*I. cylindrica* was collected from Ngopa, Mizoram, India, which is located between 23.8861° latitude north and 93.2119° longitude east. The plant specimen with voucher number PUC-I-2018-01 was authenticated at the Botanical Survey of India (BSI), Shillong, Meghalaya, India. The aerial parts were discarded and the underground parts including rhizomes and roots were dried in shade at 21–25 °C. The dried parts were ground to fine powder using electric blender.

The plant extracts were prepared in a 5-l capacity Soxhlet apparatus using three solvents such as methanol (polar solvent), chloroform (medium polar solvent) and hexane (non-polar solvent). The extracts were concentrated by removing and recovering the solvents in a vacuum rotary evaporator (Buchi Rotavapor® R-215) [14]. The final extracts were obtained as semisolid precipitates and were stored at 4 °C until use. The chloroform extract was found to contain the most abundant compounds and showed the highest biological activity, and thus was used for complete chemical analysis and anthelmintic assay.

### Chemicals and drug

All chemicals were standard analytical grades procured from HiMedia Laboratories Private Limited, Mumbai, India. Acetonitrile for gas chromatography and tetramethylsilane for electron microscopy were products of Merck Life Science Private Limited, Mumbai, India. A standard anthelmintic, albendazole (ZENTEL®) was a product of GlaxoSmithKline Pharmaceuticals Ltd., Mumbai, India.

### Chemical analysis

The chemical constituent of the plant extract was analysed in a single quadrupole gas chromatography-mass spectrometry system (Thermo Scientific TRACE™ 1300 ISQ™ LT). Acetonitrile was used to dissolve the extract. GC elution was done in a non-polar column TR-5MS (260F142P) having a dimension of 30 m × 0.25 mm × 0.25 μm and film thickness of 0.25 μm. Temperature of the injector port was set at 250 °C. The oven temperature was initially set at 70 °C for 2 min and incrementally increased at 10 °C up to 250 °C. Helium was used a carrier gas and passed at a constant flow rate of 1 ml/min. One microliter of the sample was injected in a splitting ratio of 1:50. The ionisation electron energy of the mass spectrometer was set at 70 eV. Ion source and transfer line temperature were set at 250 °C. The running duration was 55 min. The final chromatogram was generated with Thermo Scientific™ Xcalibur™ software. Compounds were identified based on their chemical formula, retention time, and molecular weight from the libraries of Wiley Registry™ and National Institute of Standards and Technology (NIST) database.

### Anthelmintic test

Anthelmintic activity was studied in vitro on the survival of tapeworm *R. tetragona*, and the roundworm *A. galli* [19]*.* The helminth parasites were recovered from the intestines of freshly sacrificed local chicken, *Gallus Gallus domesticus* Linnaeus, 1758*.* Use of chicken was permitted by the Institutional Ethics Committee of Pachhunga University College (PUC-IEC-2016-Z2 of 10/08/2016). The worms were collected and washed in Petri dishes containing 0.9% neutral phosphate-buffered saline (PBS) maintained at 37 ± 1 °C in a glass-panelled microbiological incubator. *I. cylindrica* chloroform extract was prepared in exponential concentrations of 1.25, 2.5, 5, 10 and 20 mg/ml by dissolving in PBS supplemented with 1% dimethylsulfoxide (DMSO). A broad-spectrum anthelmintic, albendazole (with a standard dosage of 20 mg/ml) was prepared similarly as a positive control. Negative control consisted of worms kept in Petri dishes containing only PBS with 1% DMSO. All the media were maintained at 37 ± 1 °C. A set of five worms were introduced into each media, and each test was performed in triplicates.

Survival was defined as a total loss of motor activity after stimulation by dipping the parasites in lukewarm PBS (45 °C). Data were generated as means ± standard deviation and presented in normalised values against the control. Student’s *t*-test was used to determine the statistical significance and the level of significance was considered when *p* value was less than 0.05.

### Scanning electron microscopy

Helminths in control experiment and those treated with 20 mg/ml of the plant extract were processed for scanning electron microscopy to compare the structural appearances based on a standardized method for helminths [20]. They were first fixed in 10% formaldehyde (buffered with 0.1 M sodium cacodylate) at 4 °C for 4 h. Then a secondary fixation was done with 1% osmium tetroxide at 4 °C for 1 h. They were dehydrated through a series of acetone in increasing concentrations. After treating with tetramethylsilane for 15 min they were dried in an air-drying chamber at 25 °C. They were mounted on metal stubs and sputter coated with gold in JFC-1100 (JEOL Ltd., Tokyo, Japan) ion-sputtering chamber. Finally, they were observed under a JSM-6360 scanning electron microscope (JEOL Ltd., Tokyo, Japan) at an electron accelerating voltage of 20 kV.

## Results

### Chemical analysis

**Fig. 1:**
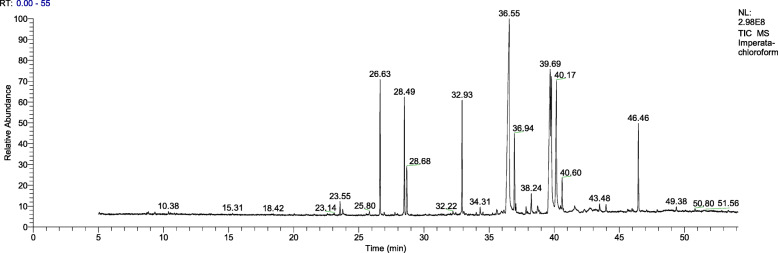
GC-MS chromatogram of the chloroform extract of *I. cylindrica* underground parts*.* Total retention time was 55 min

GC-MS chromatogram of the chloroform extract of *I. cylindrica* underground parts is shown in Fig. 1, and the corresponding list of compounds identified from it is given in Table 1. Twenty-two compounds were identified. Fatty acids are the major constituents comprise 64% of the total volatile components. Palmitic acid (hexadecanoic acid) is by far the most abundant with an abundance of 15.5%, and its methyl ester is also detected (9.5%). Other major compounds included (Z)-18-octadec-9-enolide (11.8%), 2,4-di-tert-butylphenol (11%), octadecanoic acid (10.9%), and 9-hexadecen-1-ol (907%). Phytosterols such as campesterol (1.4%) and stigmasterol (1.4%) were also found in moderate amounts.

**Table 1:** List of compounds identified from chloroform extract of *I. cylindrica* underground parts using GC-MS

Sl. no	Retention time (min)	Compound	Formula	Molecular weight (Da)	Abundance (%)
1.	10.38	3,5-Bis (1,1-dimethylethyl)-4-hydroxy benzenepropanoic acid, octadecyl ester	C_36_H_62_O_3_	530	1.1
2.	15.31	13-Heptadecyn-1-ol	C_17_H_32_O	252	1.1
3.	18.42	6-Methylenebicyclo [3.2.0] hept-3-en-2-one	C_8_H_8_O	120	1.0
4.	23.14	2-Methoxy-4-vinylphenol	C_9_H_10_O_2_	150	1.0
5.	23.55	3-(Chloroacetoxy)-4-methoxy benzaldehyde	C_10_H_9_CIO_2_	228	1.9
6.	25.80	2-Octyl cyclopropanetetradecanoic acid, methyl ester	C_26_H_50_O_2_	394	1.2
7.	26.63	2,4-Di-tert-butylphenol	C_14_H_22_O	206	11.0
8.	28.49	9-Hexadecen-1-ol	C_16_H_32_O	240	9.7
9.	28.68	2,2,4-Trimethyl-1,3-pentanediol diisobutyrate	C_16_H_30_O_4_	286	4.2
10.	32.22	(E)-4-(3-Hydroxyprop-1-en-1-yl)-2-methoxyphenol	C_10_H_12_O_3_	180	1.2
11.	32.93	Palmitic acid, methyl ester	C_17_H_34_O_2_	270	9.5
12.	36.55	Palmitic acid (hexadecanoic acid)	C_16_H_32_O_2_	256	15.5
13.	36.94	L-Methionyl-D-glutaminyl-D-methionyl-L-asparaginyl-L-lysyl-L-valyl-L-leucyl-D-α-aspartyl-L-serine	C_43_H_76_N_12_O_15_S_21_	1064	7.0
14.	38.24	Methyl 9-cis, 11 trans-octadecadienoate	C_19_H_34_O_2_	294	2.5
15.	39.69	(Z)-18-Octadec-9-enolide	C_18_H_32_O_2_	280	11.8
16.	40.17	Octadecanoic acid	C_18_H_36_O_2_	284	10.9
17.	40.60	Heptacos-1-ene	C_27_H_54_	378	3.6
18.	43.48	Octadecanoic acid, ethyl ester	C_25_H_42_O_2_	374	1.7
19.	46.46	Diisooctyl phthalate	C_24_H_38_O_4_	390	7.7
20.	49.38	17α,21β-28,30-Bisnorhopane	C_28_H_48_	384	1.5
21.	50.80	Campesterol	C_28_H_48_O	400	1.4
22.	51.56	Stigmasterol	C_29_H_48_O	412	1.4

### Anthelmintic activity

The in vitro anthelmintic activity of the chloroform extract of *I. cylindrica* against the tapeworm, *R. tetragona* is presented in Table 2. Tapeworms in the control media survived for 74.19 h. Normalised survival values indicate significant concentration-dependent effects at all concentrations tested. Albendazole was more effective and killed all the worms in 24.07 ± 1.62 h and 4.70 ± 0.84 h at the lowest (1.25 mg/ml) and highest concentrations (20 mg/ml), respectively. *I. cylindrica* chloroform extract took 91.81 ± 2.36 h and 36.53 ± 2.66 h at corresponding concentrations.

**Table 2:** Anthelmintic activity of the chloroform extract of *I. cylindrica* on *R. tetragona*

Treatment	Dose (mg/ml)	Normalised survival time (hour) in mean ± SD	***t*** value	***t*** critical value
Control	0	100.00 ± 2.00	NA	NA
Albendazole	1.25	024.07 ± 1.62*	114.35	2.05
	2.5	020.51 ± 1.21*	131.56	2.07
	5	016.56 ± 0.99*	144.85	2.08
	10	012.22 ± 1.05*	150.56	2.08
	20	004.70 ± 0.84*	170.05	2.09
*I. cylindrica* extract	1.25	091.81 ± 2.36*	010.25	2.05
	2.5	089.40 ± 1.71*	015.60	2.05
	5	082.48 ± 3.02*	018.74	2.06
	10	064.73 ± 2.55*	042.15	2.06
	20	036.53 ± 2.67*	073.85	2.06

*NA* Not applicable; *n* = 15; *t* value is the calculated difference represented in units of standard error, the higher the value, the greater the evidence against the null hypothesis; *t* critical value denotes the value that must be exceeded to get a significant level

*Significantly different at *p* < 0.05 against control

Anthelmintic activity of the chloroform extract of *I. cylindrica* on *A. galli* is shown in Table 3. Roundworms were more resilient that tapeworms and survived up to 216.29 h in control media. Albendazole was again highly effective on the roundworm. It took 43.54 ± 0.97 h and 1.81 ± 0.38 h to kill the worms at the lowest and highest concentrations, respectively. While it took 95.67 ± 1.77 h and 81.56 ± 1.71 h for *I. cylindrica* chloroform extract to kill all the worms at corresponding concentrations.

**Table 3:** Anthelmintic activity of the chloroform extract of *I. cylindrica* on *A. galli*

Treatment	Dose (mg/ml)	Normalised survival time (hour) in mean ± SD	***t*** value	***t*** critical value
Control	0	100.00 ± 0.97	NA	NA
Albendazole	1.25	043.54 ± 0.97*	159.16	2.05
	2.5	036.74 ± 0.83*	192.16	2.05
	5	026.15 ± 0.72*	236.69	2.06
	10	008.12 ± 0.92*	266.53	2.05
	20	001.81 ± 0.38*	365.24	2.10
*I. cylindrica* extract	1.25	095.67 ± 1.77*	008.30	2.07
	2.5	090.13 ± 1.63*	020.13	2.07
	5	084.46 ± 1.10*	040.99	2.05
	10	082.94 ± 2.11*	028.42	2.09
	20	081.56 ± 1.71*	036.43	2.07

*NA* Not applicable; *n* = 15; *t* value is the calculated difference represented in units of standard error, the higher the value, the greater the evidence against the null hypothesis; *t* critical value denotes the value that must be exceeded to get a significant level

*Significantly different at *p* < 0.05 against control

### Scanning electron microscopy

**Fig. 2:**
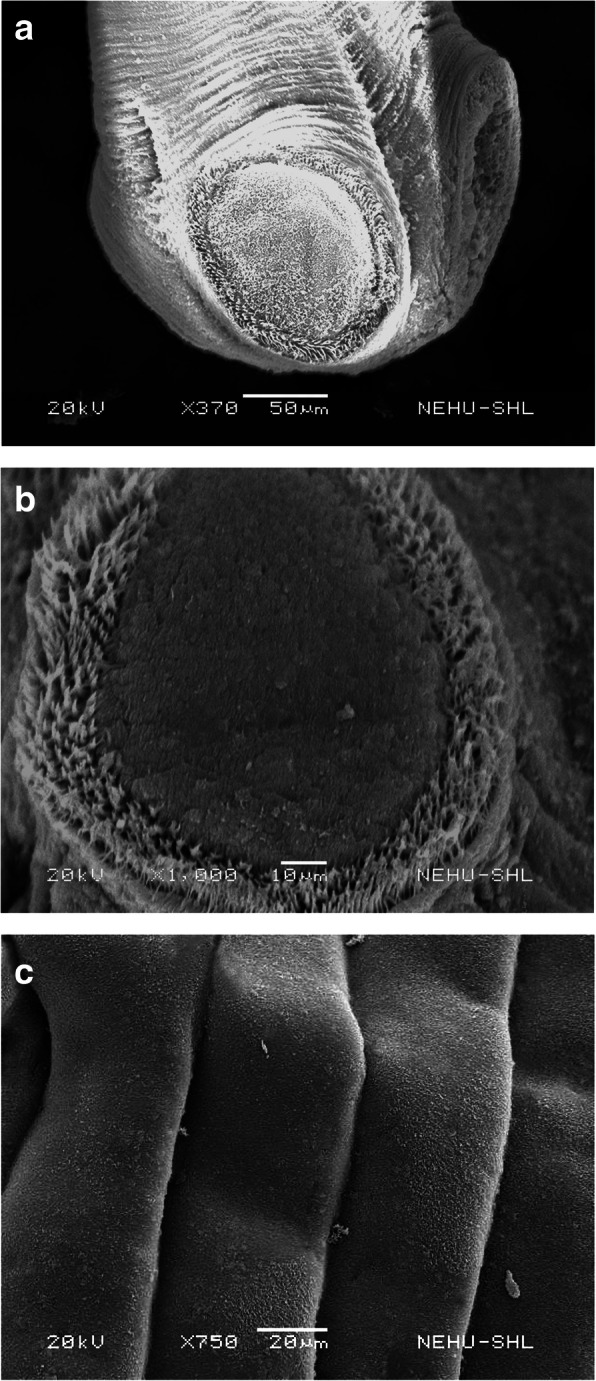
Scanning electron micrographs of an untreated *R. tetragona.* **a** The anterior end showing a bulbuous terminal called the scolex which bears prominent suckers (arrows). × 370. Scale bar = 50 μm. **b** A single sucker which is oval shaped and lined with several rows of hooks (arrows). × 1000. Scale bar = 10 μm. **c** The body segments called proglottids with smooth tegument due to fine microscopic hairs, the microtriches. × 750. Scale bar = 20 μm

Scanning electron microscopy of an untreated *R. tetragona* is shown in Fig. 2. The tapeworm has an anterior bulb-like scolex which bears the attachment organs called suckers (Fig. 2a). Each sucker is oval shaped and is lined with rows of pointed projections called the spines (Fig. 2b). The main body consists of a series of body segments or proglottids. The proglottids appear smooth and silky as the tegument (body surface) is made up of microscopic hairs called microtriches (Fig. 2c).

**Fig. 3:**
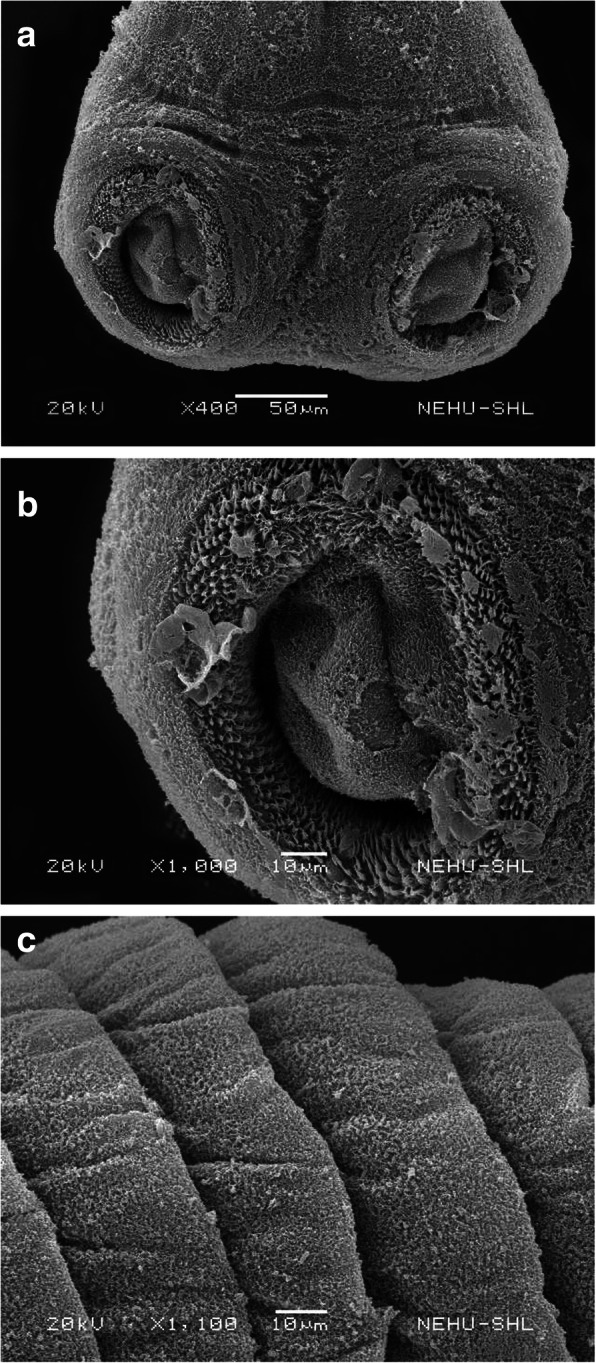
Scanning electron micrographs of *R. tetragona* treated with *I. cylindrica* extract. **a** The scolex indicating damage of tegumental (upper arrow) and suckers (lower arrow). × 400. Scale bar = 50 μm. **b** A sucker indicates clumping (upper arrow) and erosion at the central pit (lower arrow). × 1000. Scale bar = 10 μm. **c** The proglottids are wrinkled (upper arrow) and the tegument appears fuzzy due to degeneration of microtriches (lower arrow). × 1100. Scale bar = 10 μm

*R. tetragona* treated with 20 mg/ml of the chloroform extract of *I. cylindrica* revealed extensive changes on the tegument throughout the body. The scolex as shown in Fig. 3a indicates tegumental erosion and distortion of the suckers. Irregular masses on the general tegument and around the suckers are visible. In each sucker, the spines clumped and a central pit is formed which indicates tegumental erosion (Fig. 3b). Folding of the tegument and degeneration of microtriches are evident on all the proglottids (Fig. 3c).

**Fig. 4:**
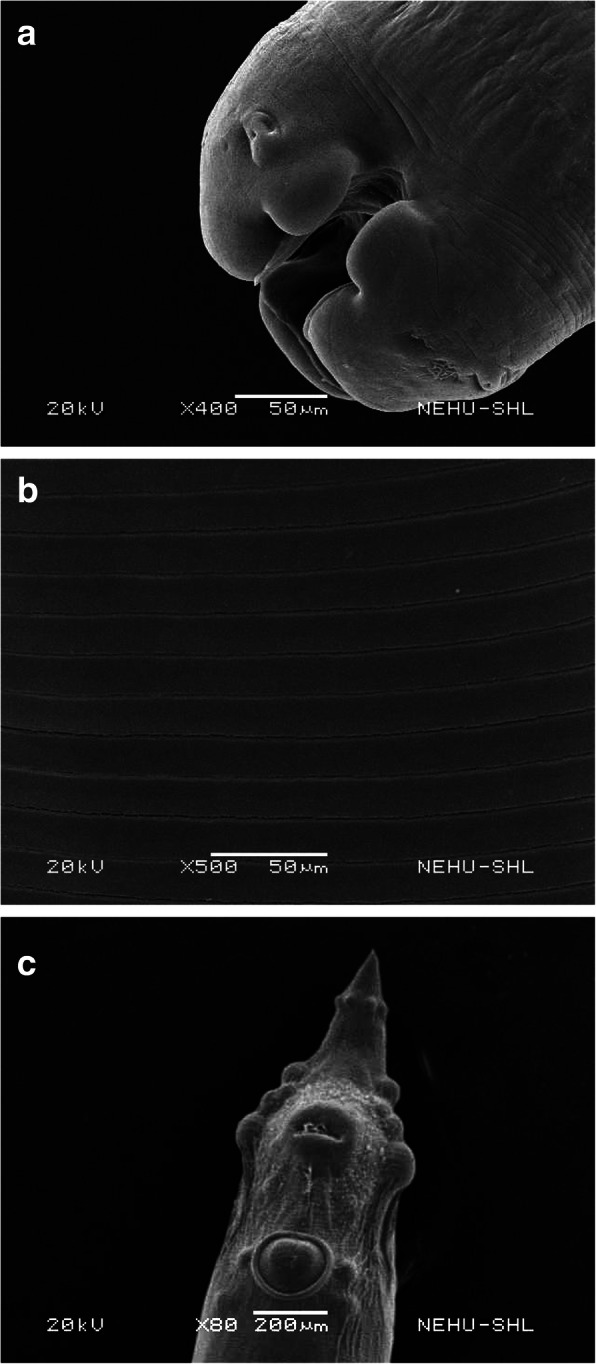
Scanning electron micrographs of an untreated *A. galli*. **a** The anterior part bears three terminal blob-like structures called the lips, which have small protrusion called papilla (upper arrow) and surround the mouth (lower arrow). × 400. Scale bar = 50 μm. **b** The body surface called the cuticle in the middle of the body is smooth and lined with parallel rows of transverse striations called annulations (arrows). × 500. Scale bar = 50 μm. **c** The posterior end is the tail which bears anal opening or cloaca (top arrow), a precloacal sucker (middle arrow) and sensory protrusions called phasmids (bottom arrow). × 80. Scale bar = 200 μm

Scanning electron microscopy of an untreated *A. galli* is presented in Fig. 4*.* The terminal end of the anterior part of the body consists of three blob-like structures called the lips. The lips surround the mouth. Sensory organs called papillae are seen as protrusions on the lips (Fig. 4a). The general body surface, called the cuticle, is rigid and smooth. There are straight and parallel transverse rings called annulations throughout the body (Fig. 4b). The tail end bears anal opening or cloaca, precloacal sucker and several sensory protrusions called amphids (Fig. 4c).

**Fig. 5:**
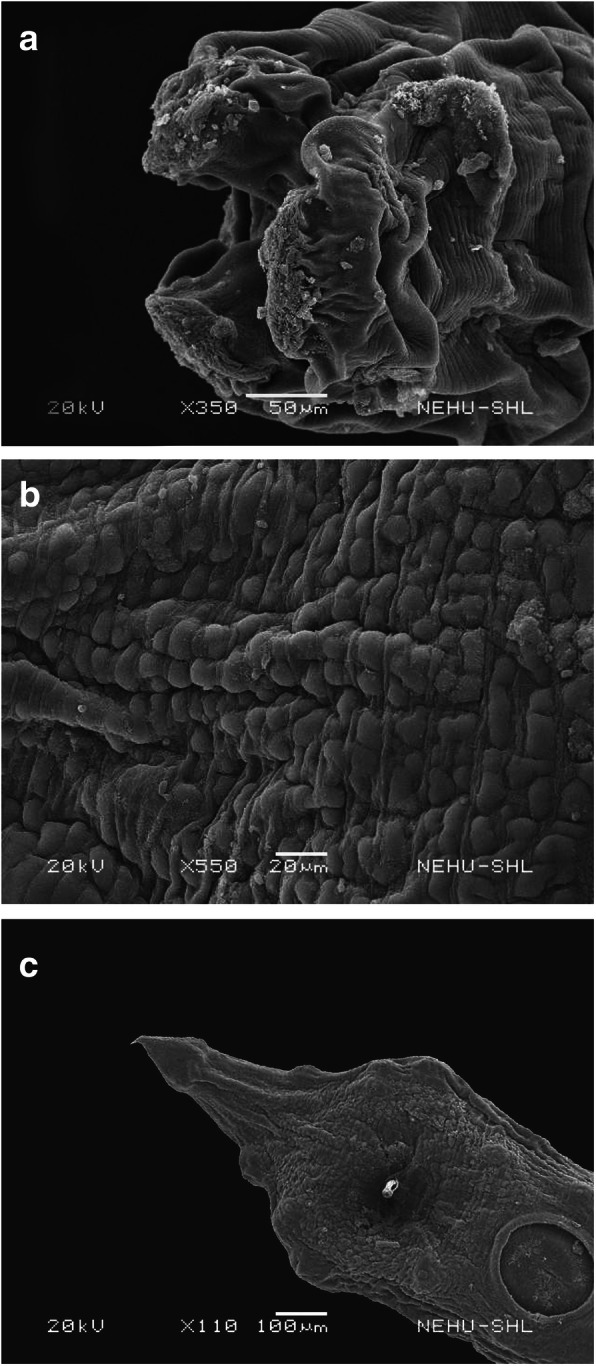
Scanning electron micrographs of *A. galli* treated with *I. cylindrica* extract. **a** The lips are shrunk and contracted (upper arrow) and the surrounding cuticle is also shrivelled (lower arrow). × 350. Scale bar = 50 μm. **b** Extensive shrinkage of the main body with formation of warts on the cuticle (arrows). × 550. Scale bar = 20 μm. **c** Shrinkage extends to the tail (top arrow), phasmids are distorted (middle arrow) and there is scar formation in the precloacal sucker (bottom arrow). × 110. Scale bar = 100 μm

The anthelmintic effects are more pronounced on *A. galli* treated with 20 mg/ml of the chloroform extract of *I. cylindrica.* The deleterious effects are particularly pronounced on the head region as shown in Fig. 5a. Three lips fully collapsed and the surrounding cuticle is massively shrunken. The main body is extensively shrunk with the formation of irregular warts all over the cuticle. The annulations are completely distorted (Fig. 5b). The deformity extends to the tail region as shown in Fig. 5c. Cuticular shrinkage extends to the tip of the tail. The amphids are degenerated and there is formation of scar in the precloacal sucker.

## Discussion

*I. cylindrica* has been an interesting plant in traditional Chinese medicine. It is known to be rich in phenols and triterpenes [21, 22]. It contains several unique compounds including graminone B [18], impecylone, impecyloside, deacetylimpecyloside, seguinoside [23], tabanone [24], and different chromones [25]. In addition, four novel chromones isolated from the rhizome exhibited neuroprotective activity in vitro [17]*.* Imperanine and cylindol A [1] and B isolated from the roots were shown to have anti-inflammatory activity [26, 27]. A novel chromone, isoegenin from the rhizome also showed anti-inflammatory activity [28].

In the present study, the rhizomes and roots appear to contain a number of important bioactive compounds. For instance, palmitic acid has antibacterial activity against different types of pathogenic bacteria [29, 30]. It also shows selective cytotoxicity upon human leukemic cells [31]. 2-Methoxy-4-vinylphenol isolated from pine (*Pinus* species) exhibits anticancer activity in vitro against different cancer cell lines [32]. It was also shown to exhibit immunomodulatory activity by enhancing antiinflammatory response suppression of NF-κB and MAPK activation, and acetylation of histone H3 [33]. 2,4-Di-tert-butylphenol from *Persea americana* is a potent antimicrobial compound against pathogenic microbes such as *Aspergillus* sp. and *Phytophthora cinnamomi* [34]. 6-Methylenebicyclo [3.2.0] hept-3-en-2-one is reported from *Allium tuberosum* and was shown to play an important role in immunity against parasitic infection such as root-knot nematode, *Meloidogyne* species [35].

Phytosterols are known for their blood-lipid lowering and anticancer activities. Campesterol inhibits fibroblast growth factor and tube formation of human umbilical vein endothelial cells, thereby indicating its role in the prevention of blood cancers [36]. Increased β-sitosterol and campesterol in the blood circulation inhibits proliferation of cancer cells in mice [37]. Campesterol, β-sitosterol, and stigmasterol are reported to decrease the risk of gastric cancer [38] and lung cancer [39]. But campesterol and stigmasterol have no beneficial effect in colon cancer [40]. These compounds are therefore likely cancer-specific and are useful for people with cardiovascular diseases and cancers [41]. Stigmasterol is further attributed to prevention of osteoarthritis as it inhibits proteins of chondrocytes [42]. It also exhibits hypoglycaemic activity by reducing serum thyroid hormones, triiodothyronine (T_3_) and thyroxin (T_4_), as well the activity of hepatic glucose-6-phophatase [43].

Substantiating the therapeutic usage of *I. cylindrica* as in the Mizo traditional medicine, the findings show that the plant extract does exert an appreciable anthelmintic activity against both tapeworm and roundworm. Damaging effects were seen on the tegument and suckers of the tapeworm *R. tetragona*, and on the cuticle and lips of the roundworm *A. galli*. These distinctive effects are to be expected because tapeworms and roundworms belong to two completely distinct classes (phyla) and have major differences in structural and physiological properties [44, 45]. In tapeworms, absorption of nutrients or drugs is directly through the body surface, the tegument, and this nature makes drugs act faster [46]. But roundworms are covered with a hard cuticle so that nutrients or drugs are absorbed poorly and slowly; thereby delaying the course of drug action [47]. A prolonged activity is therefore expected for plant extracts such as in the present study that would contain only small quantity of the bioactive compound(s). Tegumental shrinkage, disintegration of microtriches and deformity of the suckers as seen in the present study are the major effects of anthelmintic drugs on tapeworms, albeit with varying degree of damages for different drugs [48, 49]. In roundworms, the signature anthelmintic effects include shrinkage and folding of the cuticle, deflation of the lips and damages on the papillae [50, 51]. Thus, *I. cylindrica* extract produced the most notable anthelmintic effects on both tapeworm and roundworm of poultry, and thus has a potential for application in veterinary animals. However, the present in vitro test does not warrant similar efficacy for in vivo condition, therefore, poses the need for in vivo test and identification of the main anthelmintic compound.

## Conclusion

The study revealed that *I. cylindrica* rhizomes and roots contain important bioactive compounds, which exert anthelmintic activity on both tapeworm and roundworm. GC-MS analysis indicated that the chloroform extract of the underground parts is rich in saturated fatty acids. In concurrence with the usage of the plant in the Mizo traditional medicine as an anthelmintic agent, the plant extract was shown to be effective against *R. tetragona* and *A. galli.* Tegumental damages on the tapeworm and cuticular distortion on the roundworm revealed by scanning electron microscopy are clear validations of the broad-spectrum anthelmintic activity of the plant. These findings indicate that the plant contains interesting bioactive compound(s) that can serve as lead molecule in anthelmintic development.

## Data Availability

The datasets during and/or analysed during the current study are available from the corresponding author on reasonable request.

## Abbreviations

BSI: Botanical Survey of India
DMSO: Dimethylsulfoxide
GC-MS: Gas chromatography-mass spectrometry
HIV: Human immunodeficiency virus
MAPK: Mitogen-activated protein kinase
NIST: National Institute of Standards and Technology
NF-κB: Tumour necrosis factor kappa B
PBS: Phosphate-buffered saline
SD: Standard deviation
T_3_: Triiodothyronine
T_4_: Thyroxin
